# Errata

**DOI:** 10.1289/ehp.121-a73

**Published:** 2013-03-01

Feurstein et al. have reported errors in their paper “Investigation of Microcystin Congener–Dependent Uptake into Primary Murine Neurons” [Environ Health Perspect 118:1370–1375 (2010)]: In “Materials and Methods” under “PP [protein phosphatase] activity after exposure to single MC congeners” (p. 1371), some of the concentrations given for enzyme solution buffer and reaction buffer represent stock concentrations instead of final concentrations.

The corrected text is as follows:

Cells were exposed to varying concentrations of single MC congeners for 48 hr and solubilized in 50 µL enzyme solution buffer [52 mM Tris-HCl (pH 7.0), 0.1 mM ethylene glycol tetraacetic acid, 1 mM dithiothreitol (DTT; in 0.01 M sodium acetate, pH 5.2), 2 mM manganese chloride (MnCl_2_)]. Protein concentrations were determined using the method of Bradford (1976). Subsequently, 20 µL of each sample (containing 10 µg total protein) was transferred to a 96-well plate, and an equal volume of water was added. To determine PP activity, 200 µL freshly prepared and prewarmed (37°C) substrate solution containing reaction buffer [125 mM Tris-HCl (pH 8.1), 0.4 mM MnCl_2_, 52 mM magnesium chloride, 1 mg/mL bovine serum albumin (BSA)], 60 mM *p*-nitrophenyl phosphate (pNPP; Acros Organics, Morris Plains, NJ, USA), and 2 mM DTT were added and immediately measured at 405 nm (TECAN infinite M200; TECAN, Craislheim, Germany) to determine the 0-time value.

These changes do not affect the results or conclusions of the paper.

The authors regret the errors.

The January news article “Beyond Uncertainty Factors: Protecting the Tails of the Bell Curve” [Environ Health Perspect 121:A26–A29 (2013)] incorrectly cited the 1997 book *Our Stolen Future* as the source of the figures and related quotation on p. A28. The figures and quotation actually came from the website http://www.ourstolenfuture.org/ and were developed by John Peterson Myers, a coauthor of the 1997 book. However, they did not appear in the book itself.

*EHP* regrets the error.

